# MRE11:p.K464R mutation mediates olaparib resistance by enhancing DNA damage repair in HGSOC

**DOI:** 10.1186/s13578-023-01117-0

**Published:** 2023-09-27

**Authors:** Xucui Zhuang, Rourou Xiao, Yu Fu, Bin Yang, Junpeng Fan, Funian Lu, Tianyu Qin, Xiaohang Yang, Xingyuan Hu, Jingjing Yin, Wenting Li, Xiaoyan Kang, Gang Chen, Dianxing Hu, Chaoyang Sun

**Affiliations:** 1 Department of Gynecological Oncology, Tongji Hospital, Tongji Medical College, Huazhong University of Science and Technology, Wuhan, China; 2 National Clinical Research Center for Obstetrics and Gynecology, Cancer Biology Research Center (Key Laboratory of the Ministry of Education), Tongji Hospital, Tongji Medical College, Huazhong University of Science and Technology, Wuhan, China

**Keywords:** MRE11:p.K464R mutation, Non-homologous end joining, Olaparib resistance, Ovarian cancer

## Abstract

**Background:**

Although the clinical application of PARP inhibitors has brought hope to ovarian cancer, the problem of its resistance has become increasingly prominent. Therefore, clinical experts have been focused on finding specific indicators and therapeutic targets that can be used for resistance monitoring of PARP inhibitors.

**Results:**

By cfDNA detecting during Olaparib maintenance therapy in platinum-sensitive relapsed ovarian cancer, we found the presence of MRE11:p.K464R mutation was strongly associated with acquired Olaparib resistance. Structural analysis revealed that the MRE11:p.K464R mutation is situated at a critical site where the MRE11 protein interacts with other biomolecules, leading to potential structural and functional abnormalities of MRE11 protein. Functionally, MRE11:p.K464R mutation enhanced the tolerance of Olaparib by reducing the DNA damage. Mechanistically, MRE11:p.K464R mutation improved the efficiency of DNA damage repair and induce Olaparib resistance by enhancing its binding activity with the interacting proteins (including RAD50 and RPS3). Among them, the enhanced binding of MRE11:p.K464R mutation to RAD50/RPS3 facilitated non-homologous end joining (NHEJ) repair in tumor cells, thereby expanding the scope of research into acquired resistance to PARP inhibitors.

**Conclusions:**

Our findings provide a theoretical basis for MRE11:p.K464R mutation as a specific indicator of resistance monitoring in Olaparib treatment, and the exploration of its resistance mechanism provides a novel insights for the formulation of combination ther therapies after Olaparib resistance.

**Supplementary Information:**

The online version contains supplementary material available at 10.1186/s13578-023-01117-0.

## Introduction

High Grade Serous Ovarian Cancer (HGSOC) is the most common pathological type of Ovarian epithelial tumors, with the highest mortality rate among gynecological tumors. In advanced patients, the 5-year survival rate is less than 30% [1]. Although surgical treatment and paclitaxel plus platinum-based chemotherapy have brought some relief to patients, 70–80% of them will still relapse, and the relapsed patients are more likely to develop platinum resistance [2, 3]. Therefore, clinical experts have been exploring ways to reduce the recurrence of ovarian cancer and utilize them as maintenance therapy options.

In recent years, the clinical application of PARP inhibitors has brought new light to ovarian cancer patients, which have shown significant long-term survival benefits in a number of clinical studies, such as SOLO-1, PRIMA and PAOLA-1 [4]. However, with the widespread use of PARP inhibitors, the risk of drug resistance has become increasingly prominent. Understanding the mechanisms underlying PARP inhibitor resistance is crucial for devising precise treatment strategies and overcoming treatment resistance. Unfortunately, although existing studies have explored the mechanisms of PARP inhibitor resistance from multiple perspectives [5], including homologous recombination repair (HRR) function restoration (BRCA1/2 reversion mutation [6–8] and abnormal DNA repair proteins [9–11]), DNA replication fork protection [12], PARP function loss [13], etc., none of them offered definitive solutions to improve the treatment outcomes for patients. Moreover, there is still a lack of specific and accurate indicators for evaluating the efficacy and monitoring resistance to PARP inhibitors. To solve the clinical dilemma of PARP inhibitor resistance, it is urgent to screen the molecular characteristics related to drug resistance with potential clinical translational value.

The MRE11-RAD50-NBS1 (MRN) complex plays an important role in HRR after DNA damage, and alterations in its structure and function are closely associated with chemotherapy resistance. It has been reported that the upregulation of MRN complex can enhance the efficiency of DNA damage repair and mediate DNA damage-based tumor therapy resistance [14, 15]. MRE11, as the core component of MRN complex, is recruited to the damage site when DNA double-strand breaks and initiates the HRR [16]. The abnormality of MRE11 protein inevitably leads to changes in the structure and function of MRN complex. In the previous study, we found the high-frequency mutation of MRE11:p.K464R in circulating cell-free DNA of ovarian cancer patients with Olaparib resistance and observed a significant concomitant relationship between MRE11:p.K464R mutation and disease progression, which strongly suggested that MRE11:p.K464R mutation may induce the development of Olaparib resistance [17]. Therefore, we hypothesized that MRE11:p.K464R mutation enhances the efficiency of DNA damage repair and mediates the development of resistance to PARP inhibitors.

In this study, we investigated the impact of MRE11:p.K464R mutation in Olaparib resistance in a variety of ovarian cancer cell lines and elucidated the underlying mechanisms of resistance. Further, we investigated the possibility of reversing Olaparib resistance in ovarian cancer patients caused by MRE11:p.K464R mutation through targeted intervention of related molecules, including RAD50 and RPS3. Our results laid a theoretical foundation for MRE11:p.K464R mutation as a reliable indicator for resistance monitoring of Olaparib, which is expected to improve the prognosis of patients and promote the progress of diagnosis and treatment of ovarian cancer patients.

## Materials and methods

### Cell culture

Human ovarian serous adenocarcinoma cell lines SKOV3 (ATCC® HTB-77™) were obtained from the American Type Culture Collection (ATCC, Manassas, VA, USA). Human ovarian carcinoma cell lines A2780 were obtained from the M.D. Anderson Cancer Center characterized Cell line Core (Houston, TX, USA). The cells were cultured in RPMI-1640 medium (Gibco), supplemented with 10% fetal bovine serum (FBS, BI) and 1% penicillin/streptomycin (Gibco) at 37 °C in a humidified atmosphere containing 5% CO2.

### Construction of MRE11 knockout cell lines

The CRISPR-Cas9 system was used to construct MRE11-KO cell lines (SKOV3-MRE11^KO^ and A2780-MRE11^KO^). Design and sequence of sgRNA: the CRISPick platform (https://portals.broadinstitute.org/gppx/crispick/public) was used for the design of sgRNA, and the optimal three sequences were selected for synthesis and vector construction; the optimal sequence was selected by knockout efficiency verification for subsequent experiments (sgRNA forward: 5’-CACCGGTTTGCTGCGTATTAAAGGG-3’, sgRNA reverse: 5’-AAACCCCTTTAATACGCAGCAAACC-3’). Construction of plasmids: the sgRNA sequences were synthesized by Tsingke Biotechnology Co., Ltd. (Beijing, China); after the vector plasmid was digested with the restriction endonuclease BsmBIv2 (CAT#0739S, NEBiolabs), the sgRNA was ligated to the pLentiCRISPR v2 vector by T4 DNA ligase (CAT#2011A, Takara); the ligation product was transferred into DH-5α competent cells, and single clones were picked and confirmed by DNA sequencing. Preparation of Lentivirus: HEK293T cells were co-transfected with pCMV-△8.91 lentivirus packaging plasmid, pcMV-VSVG envelope plasmid and pLentiCRISPR v2 vector or pLentiCRISPR V2-sgRNA plasmid, respectively; after 6-8 h of transfection, the medium was changed to fresh medium; after 48 h, the supernatant viral was collected, filtered through a 0.45 μm filter to remove particle residues, and stored at -80 ℃ until use. Infection of Lentivirus: SKOV3 and A2780 cells were seeded in six-well plates; after the cells had grown to 30–40%, 2 mL of virus suspension was mixed with 1 mL of complete medium, supplemented with 10 ug/mL ploybrene (CAT#40804ES76, YEASEN), and added; after 12–16 h of transfection, the medium was changed to fresh medium, after 48 h of infection, 2 ug/ mL puromycin was added to screen for stable cell lines. Validation of MRE11 knockdown cell lysates and genomic DNA were collected, and MRE11 knockdown efficiency was identified by WB and DNA assays, respectively.

### Overexpression of MRE11

A lentiviral vector carrying mCherry (CV572, Shanghai GeneChem Co., Ltd., Shanghai, China) was used to overexpress MRE11^WT^ or MRE11^K464R^ protein. The lentiviral vector that expresses mCherry alone was used as a control. 6–10*10^4 SKOV3-MRE11^KO^ and A2780-MRE11^KO^ cells were seeded in six-well plates for 2 4 h, 1 ml of complete medium with appropriate virus suspension (SKOV3 MOI = 20, A2780 MOI = 10), supplemented with 10 ug/mL ploybrene (CAT#40804ES76, YEASEN), and added, after 12–16 h of transfection, the medium was changed to fresh medium. After 48 h of infection, G418 (CAT#A2513, ApExBIO) (SKOV3 400 ug/ml, A2780 200 ug/ml) was added to screen for stable cell lines, and the fluorescence expression (mCherry) of cells was observed to evaluate the virus infection efficiency and cell lysates were collected to verify MRE11 protein expression by WB, including MRE11^WT^ and MRE11^K464R^. Cell clones expressing MRE11 protein were collected and expanded for further studies.

### RNA interference

All siRNAs employed in this study were synthesized by Tsingke Biotechnology Co., Ltd. (Beijing, China) and listed in Table S1. RNA interference transfections were performed according to the procedure for Lipofectamine 3000 Transfection Reagent (CAT#L3000015, Thermo Fisher Scientific). Cell lysates were collected to assess the efficiency of RNA interference by WB.

### Cell viability assay

Cell Counting Kit-8 analysis (CAT#CK04, Dojindo Laboratories) was used for detecting the cell viability. In brief, 3000–5000 (depending on cells) cells were seeded in 96-well plates for 24 h, followed by drug administration for 72 h. Then the media were carefully replaced with 100 ul fresh media containing 10% detection reagent in the dark. After incubation for 2 h at 37 ℃ in a humidified incubator with 5% CO2, the plates were measured at the absorbance of 450 nm using a microplate reader (Bio-Rad, CA, USA). Relative cell viability was calculated using the control as a reference. The experiments were repeated three times and statistics were performed using GraphPad Prism 8.0.

### Clone formation assay

The cells were seeded in 6-well plates at a density of 1000 cells/well and treated with drugs for 7–10 days (depend on cells) after cell attachment. Cells were then fixed using 4% paraformaldehyde, stained with crystal violet for 10 min. Excess dye was washed off and completely dried before imaging. The number of clones in different experiment groups was counted and compared.

### Transwell assay

Cell migration ability was examined using 24-well transwell chambers (pore size, 8 μm; Costar, Cambridge, USA). 3 × 10^4^ SKOV3 or 5 × 10^4^ A2780 cells, respectively, were seeded on the upper chamber while complete medium with were placed in the lower chamber. After 24 h, the inserts were removed and migrated cells were stained with 0.05% crystal violet.

### Wound healing assay

Cells were taken and placed in a six-well plate with a cell density of 5 × 10^5^/well. When the cells were adherent to the wall in a single layer, a pipetting tip of 200 µL was used to vertically scratch the six-well plate, and cleaned the suspension cells with PBS. Cells were cultured in an incubator with 5% CO2 at 37 °C. Photographs were taken at 0 h, 12 and 24 h under the microscope.

### Western blots

Cells were lysed on the ice for 15 min with RIPA buffer (CAT #G2002; Servicebio) supplemented with 1% PMSF and 2% cocktail. Lysates were lysed on ice for 10 min after sonication, followed by centrifugation at 12,000 g for 15 min at 4 ℃, and the supernatant was collected for further studies. Nuclear protein was extracted using Nuclear and Cytoplasmic Protein Extraction Kit (CAT#P0027, Beyotime). For Chromatin fractionation, we used a Subcellular Protein Fractionation kit (CAT#78,840, Thermo) following the manufacturer’s instructions. Protein concentration was measured using Coomassie (CAT#ST1119, CAT#P0006C, Beyotime). The loading amount (20–40 ug) was determined according to the expression of the target protein. Proteins were separated in 10% SDS-PAGE gels (CAT#8,012,011, BioSci), and electro-transferred onto polyvinylidene fluoride membranes (CAT #10,600,023, PVDF, Cytiva Life Sciences). The PVDF membrances were blocked using 5% BSA for 1 h and incubated with the following primary antibody at 4 ℃ overnight: MRE11 (CAT#4895, CST), RAD50 (CAT#3427, CST), RPS3 (CAT#66046-1, Proteintech), γH2AX (CAT#AP0099, ABclonal), LaminB1 (CAT#A11495, ABclonal), DNA-PKcs (CAT#A20837, ABclonal), DNA Ligase IV (CAT#A11432, ABclonal), KU70 (CAT#A11223, ABclonal), pCHK1 Ser345 (CAT#2348,CST), pCHK2 Thr68 (CAT#2197,CST), pATM Ser1981 (CAT#2348,CST), pATR Ser428 (CAT#2853,CST), β-actin (CAT#AC026, ABclonal), followed by incubation with 1:5000 secondary antibodies (CAT#AS014 and CAT#AS003, Abclonal) for 1 h at room temperature. Bands were visualized in a ChemiDoc Imaging System (Bio-Rad, CA, USA) using the ECL Western Blotting Substrate (CAT#K-12,045-D50, Advansta).

### Immunoprecipitation-mass spectrometry

To identify the interacting proteins of MRE11 in cells, MRE11 and its interacting proteins were extracted by IP kit (CAT#abs955, Absin) and analyzed by mass spectrometry. Mass spectrometry data analysis was performed as reported in previous studies. In brief, the database search was carried out on the raw data to identify the species and relative quantitative information of proteins in the samples. Next, the quantitative data were preprocessed, including data conversion, invalid data elimination, etc. Compared with the WT group, the higher proteins (Fold Change > 2) were defined as the highly credible interacting proteins of MRE11:p.K464R in the K464R group. Finally, the direct interacting proteins of MRE11:p.K464R were clarified using the STRING database [18].

### Cellular immunofluorescence assay

Cells were seeded onto 12-mm glass slides in a 24-well plates, and Olaparib treatment was given at 70% confluence. The glass slides with cells were collected after being treated for 48 h, followed by washed with PBS, fixed with 4% polyoxymethylene for 30 min, and permeabilized with 0.3% Triton X-100 for 15 min. Next, the glass slides were washed again with PBS, blocked with 5% FBS for 30 min at room temperature and incubated with the primary antibody at 4 ℃ overnight, including MRE11 (CAT#4895, CST), RAD50 (CAT#GTX70228, GeneTex), γH2AX (CAT#201082-2A9, ZENBIO), DNA-PKcs (CAT#A20837, ABclonal), DNA Ligase IV (CAT#A11432, ABclonal), KU70 (CAT#A11223, ABclonal). The secondary antibody of Alexa Fluor 488 donkey anti-mouse IgG (CAT#ANT023s, Antgene, 1:200), Alexa Fluor 488 donkey anti-rabbit IgG (CAT#ANT024s, Antgene, 1:200), Alexa Fluor 594 donkey anti-rabbit IgG (CAT#ANT030s, Antgene, 1:200) and Alexa Fluor 647 donkey anti-mouse IgG (CAT#A-21,236, Thermo, 1:1000) were performed for 1 h at room temperature. The glass slides were sealed using mounting medium containing DAPI. Cells were observed and photographed using the following equipment: fluorescence, NIKON Eclipse Ti; software: Eclipse C2, Nikon. Image-pro Plus 6.0 was used for image analysis.

### Comet assay

Alkaline comet assays were performed using the Comet Assay kit (CAT#4250-050-k, Trevigen) according to the manufacturer’s instructions. In short, the treated cells were mixed with LM agarose at 37 ℃ and spread on a Comet Slide. The slides were left at 4 ℃ in the dark for 30 min, lysed at 4 ℃ for 1 h in the lysis solution, and then placed them in the unwinding solution (pH > 13) for 20 min at room temperature in the dark. Electrophoresis was carried out at 4 ℃ in alkaline electrophoresis solution with 21 V voltage for 30 min, fixed with 70% ethanol and stained with SYBR Green. The comets were observed and photographed using the following equipment: fluorescence, NIKON Eclipse Ti; software: Eclipse C2, Nikon. At least 50 cells were measured for each sample, and the average damage from three independent experiments was calculated.

### NHEJ reporter assay

EJ5-Hela cells were transfected with the indicated plasmids or siRNAs for 24 h, and then transfected with the I-SceI expression plasmid or infected with the retrovirus expressing I-SceI. After 48 h recovery, the cells were analyzed by FACS. At least three biological repeats were performed.

### Chromatin immunoprecipitation (ChIP-qPCR)

ChIP assays were performed with Enzymatic Chromatin IP kit (CAT#9003, CST) as described in manufacturer’s instructions. The ER-AsiSI Hela cells transfected with the indicated plasmids or siRNAs for 24 h, and then treated with 4-hydroxytamoxifen (4-OHT; CAT#HY16950, MCE) to induce DSBs [19]. Next, cells were cross-linked with 1% formaldehyde and neutralized with 125-mM glycine. The cross-linked nuclear lysates were digested with micrococcal nuclease, and then sonicated to yield genomic DNA fragments between 150 and 900 bp. The digested chromatin was immunoprecipitated with the indicated primary antibody overnight at 4 °C. Anti-RAD50 antibody (CAT#GTX70228, GeneTex), anti-RPS3 antibody (CAT#66046-1, Proteintech), anti-KU70 antibody (CAT#66607-1, Proteintech) or normal Rabbit IgG control were added to chromatin samples, followed by overnight incubation at 4 °C with rotation. Antibody-chromatin complexes were captured using magnetic protein A/G beads. Purified DNAs were subjected to quantitative PCR (qPCR). The sequences of qPCR primers are shown in Supplementary Table S1.

### In situ proximity ligation assay (PLA)

Cells were grown on coverslips, washed with PBS and fixed with 0.1% paraformaldehyde for 5 min, followed by two treatments totaling 10 min with CSK-R buffer (10 mM PIPES, pH 7.0, 100 mM NaCl, 300 mM sucrose, 3 mM MgCl2, 0.5% Triton X-100, 300 µg/ml RNase), and fixed in 4% paraformaldehyde in PBS (W/V) for 10 min at RT, followed by incubation in pre-cold methanol for 20 min at -20 °C. After washing with PBS for three times, cells were treated with 100 ug/ml RNase in 5 mM EDTA buffer for 30 min at 37 °C. In situ PLA was performed using the Duolink PLA kit (Sigma-Aldrich) according to the manufacturer’s instructions. Briefly, the cells were blocked for 60 min at 37 °C and incubated with primary antibody overnight at 4 °C (MRE11 antibody (CAT#4895, CST), RAD50 antibody (CAT#GTX70228, GeneTex) or RPS3 antibody (CAT#66046-1, Proteintech). The next day after washing with PBS twice, cells were incubated with pre-mixed PLA probe anti-mouse minus and PLA probe anti-rabbit plus (Sigma) for 1 h at 37 °C. After three times washing with buffer A for 5 min, PLA probes were ligated for 30 min at 37 °C. After three times washing with buffer A, amplification using Duolink In Situ Detection Reagents (Sigma) was performed at 37 °C for 100 min. After amplification, the plates were washed for 5 min three times with wash buffer B and one time with 0.01× buffer B and then stained with DAPI and imaged on Olympus Laser Scanning Confocal Microscopy at ×100.

### Statistical analysis

Unpaired two-tailed Student’s t test was used for analyzing the significant differences between two groups. For comparisons of multiple groups, ordinary one-way ANOVA with Tukey’s multiple comparisons test was used. Log-rank (Mantel-Cox) test was utilized to determine the differences in survival curves. The above statistics were analyzed and plotted using GraphPad Prism 8 (GraphPad Software). Results were considered statistically significant when p < 0.05.

## Results

### Acquired MRE11:p.K464R mutation is strongly associated with resistance to olaparib maintenance treatment in platinum-sensitive relapsed ovarian cancer

**Fig. 1:**
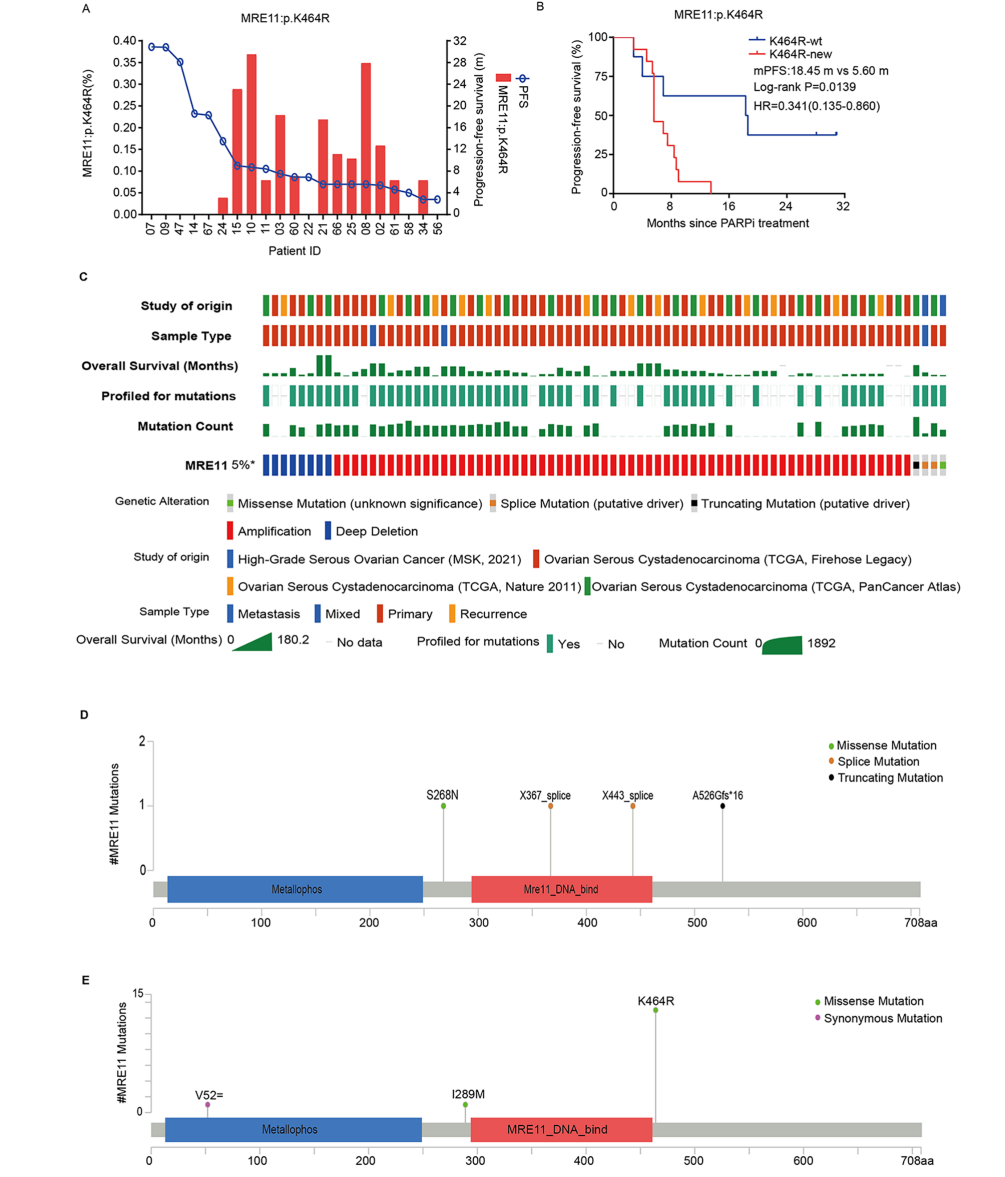
**Association between acquired MRE11:p.K464R mutation and the prognosis of ovarian cancer patients**. (**A**) The distribution of MRE11:p.K464R mutation content (cfDNA) and PFS in patients. (**B**) Survival analysis of acquired MRE11:p.K4 mutation in patients. (**C**) The variations of MRE11 in four serous ovarian cancer datasets of cBioportal database. (**D**) The distribution of MRE11 mutation types in four serous ovarian cancer datasets. (**E**) The distribution of MRE11 mutation types in this study

To elucidate the role of tumor mutations in the development of acquired resistance to PARP inhibitors and to screen for specific mutational markers associated with resistance, we examined the mutation profiles in cfDNA of ovarian cancer patients during Olaparib maintenance therapy [17]. We found that acquired MRE11:p.K464R mutation was strongly related to Olaparib resistance. Firstly, we plotted the distribution relationship between MRE11:p.K464R mutation content and progression-free survival (PFS) of patients, which showed that the acquisition of this mutation had an obvious concomitant relationship with the shortened PFS in patients (Fig. 1A). Further survival analysis revealed that acquired MRE11:p.K464R mutation significantly reduced the survival benefit of patients during Olaparib maintenance therapy (Fig. 1B), suggesting that the K464R mutation in MRE11 protein may play a critical role in driving resistance to Olaparib in patients. To further clarify the specificity of acquired MRE11:p.K464R mutation, we mined four serous ovarian cancer datasets in cBioportal database (www.cbioportal.org) [20, 21] and found that the variants of MRE11 gene had been rarely reported in previous studies, with a mutation frequency of about 5% in the population, and only four patients had mutations in MRE11 (Fig. 1C). Unfortunately, we did not observe the MRE11:p.K464R carriers by comparing the mutation types (Fig. 1D). While differences in the detection objectives of various datasets may impact the detection rate of MRE11 mutations, the occurrence of acquired MRE11:p.K464R high-frequency mutation during Olaparib maintenance therapy in this study has extremely high specificity and accuracy (Fig. 1E). Therefore, it is reasonable to believe that the acquisition of MRE11:p.K464R mutation in tumor cells mediates the development of resistance to Olaparib.

### Acquired MRE11:p.K464R mutation leads to structural abnormality of MRE11 protein

**Fig. 2:**
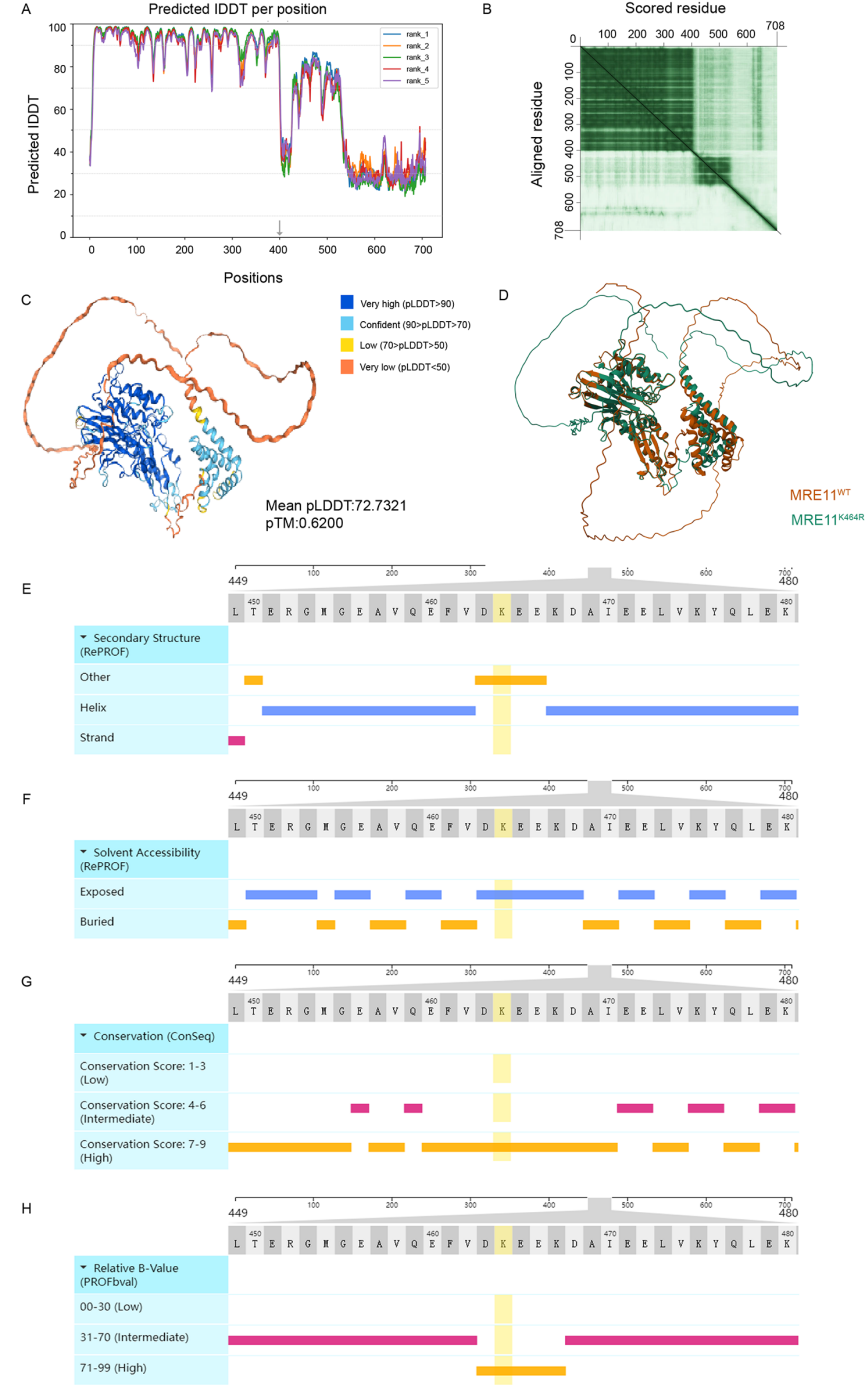
**Effects of K464R mutation on the structure of MRE11 protein**. (**A**) The per-residue confidence score using AlphaFold 2 algorithm (Rank 1–5). (**B**) The predicted 3D structure based on the Rank 1 model confidence (pLDDT). (**C**) The display of Predicted Alignment Error (PAE) based on the Rank 1 model. (**D**) The comparison of 3D structures between MRE11WT and MRE11K464R predicted by AlphaFold 2 algorithm. (**E**) The position of the K464R mutation site in the secondary structure of MRE11 protein. (**F**) The K464R mutation site is located in the exposed region of solvent accessibility. (**G**) The amino acid sequence at the position of the K464R mutation is highly conserved. (**H**) The position of the K464R mutation site has a high degree of protein flexibility (a high relative B-value)

Considering the novelty of acquired MRE11:p.K464R mutation, we attempted to analyze the influence of K464R mutation on the three-dimensional (3D) structure of MRE11 protein by protein structure prediction. The results of 3D structure prediction using Alpha Fold 2 algorithm (https://colab.research.google.com/github/sokrypton/ColabFold/) showed that the average pLDDT (predicted local distance difference test) of the region where K464R site was located was low, and there were predicted sites with very low confidence before and after it (Fig. 2A, **Rank1-5;** Fig. 2C, Rank 1; supplement Fig. S1A-D). In addition, the PAE (Predicted Aligned Error) of the 400aa-708aa in MRE11 protein were large (Fig. 2C; http://www.subtiwiki.uni-goettingen.de/v4/paeViewerDemo). These results suggested that accurate 3D predicted structures could not be obtained. Similarly, our prediction results based on the other three algorithms (including (I-TASSER [22–24], tFold [25] and Swiss-model [26]) also supported the result that the 400aa-708aa region of MRE11 protein exhibited lower prediction confidence, with significant discrepancies in the structure predictions (supplement Fig. S1E). However, the predicted structural comparison between MRE11_WT and MRE11:p.K464R indicated that the K464R mutation is likely induce a conformational change in the C segment of the MRE11 protein (Fig. 2D;https://www.rcsb.org/alignment).

Therefore, we further analyzed the possible effect of the K464R mutation site on the peptide chain structure of MRE11 protein. The secondary structure, solvent accessibility, conservation and structural flexibility were analyzed utilizing the ProNA2020 platform (www.predictprotein.org) [27]. The results showed that the K464R mutation site was located at the helix-strand junction in the secondary structure (Fig. 2E), which could affect the spatial orientation and stability of the peptide chain. In terms of solvent accessible, the amino acid of K464R mutation site was in the exposed state, representing an important binding site for biomolecules (Fig. 2F). Sequence conservation analysis showed that this mutation site was in the conserved region of MRE11 protein (Fig. 2G), which indicated that the region where the mutation site was located had important biological functions. Structural flexibility analysis further revealed that the K464R mutation was an important functional site of MRE11 protein (Fig. 2H). In summary, the K464 site of MRE11 protein is an important biomolecular binding site, and its mutation can easily lead to structural and functional abnormalities of MRE11 protein.

### MRE11:p.K464R mutation lead to Olaparib resistance in ovarian cancer cells

**Fig. 3:**
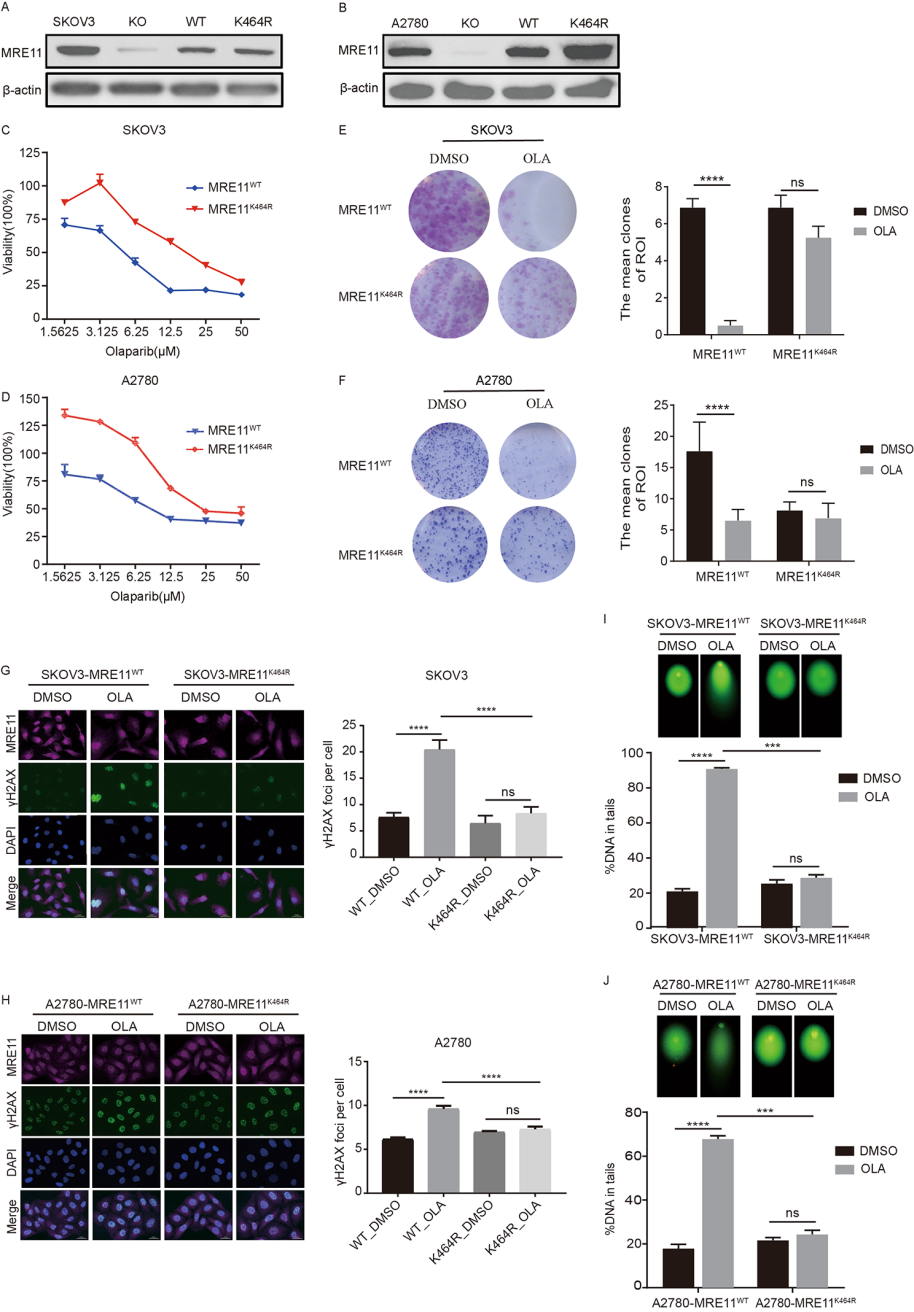
**MRE11:p.K464R mutation leads to Olaparib resistance in ovarian cancer cells**. (**A**-**B**) The stable cell lines of SKOV3 (**A**) and A2780-MRE11^WT^/MRE11^K464R^ (**B**) were constructed expressing MRE11^WT^ and MRE11^K464R^ protein and detected the MRE11 protein expression with Western blot. (**C**-**D**) SKOV3 (**C**) and A2780 (**D**) MRE11^WT^ /MRE11^K464R^ cells were treated for 96 h with indicated dose of Olaparib and viability assessed by CCK8. (E-F) Representative pictures of colony formation assay in SKOV3 (**E**) and A2780 (**F**) MRE11^WT^ /MRE11^K464R^ cells treated with or without Olaparib for 10 days are in left. Relative colony formation rates of cell are presented as percent relative to DMSO (right). (**G**-**H**) SKOV3 (**G**) and A2780 (**H**) MRE11^WT^/MRE11^K464R^ cells were treated with or without Olaparib for 48 h and then stained for γH2AX, MRE11, and DAPI (left), and quantified the γH2AX foci per cell (right). Scale bar, 25 μm. (**I**-**J**) SKOV3 (**I**) and A2780 (**J**) MRE11^WT^/ MRE11^K464R^ cells were treated with or without Olaparib for 48 h and subjected to Comet analysis. DNA damage is quantified as percent DNA in tails. Each group represents at least 100 cells counted. Data are presented as mean values ± SEM from three independent experiments. ***p < 0.001, ****p < 0.0001, ns, not significant, as determined by the unpaired two-tailed Student’s t-test

In order to further clarify the functional phenotype of Olaparib resistance caused by MRE11:p.K464R mutation, we selected two ovarian cancer cell lines (SKOV3 and A2780) and generated stable cell lines expressing MRE11^WT^ and MRE11^K464R^ protein (Fig. 3A and B) after MRE11 knockout (Fig. S1A and S1B), respectively. Subsequently, we analyzed the effect of MRE11:p.K464R mutation on cell phenotype. The results showed that the acquisition of this mutation significantly enhanced the ability of cell invasion and metastasis, which was manifested as accelerated wound healing rate (Fig. S1C and S1D) and enhanced cell migration ability (Fig. S1E and S1F). Furthermore, by drawing resistance curves and colony formation experiments, we observed significant drug resistance to Olaparib in both K464R mutant cell lines, and they exhibited a higher IC50 threshold than wild-type (WT) cells (Fig. 3C and D) and had a more pronounced proliferation advantage under the same drug concentration (Fig. 3E F). Due to the DNA damage effect mediated by Olaparib, we examined the distribution of γH2AX, a DNA damage marker, in different treatment groups by cellular immunofluorescence assay, and the results showed that Olaparib treatment significantly increased the content of γH2AX in WT cells, but the acquisition of MRE11:p.K464R mutation partially reversed the accumulation of DNA damage induced by Olaparib (Fig. 3G H). In addition, using comet assay, we further demonstrated that MRE11:p.K464R mutation enhanced the resistance of ovarian cancer cells to Olaparib and reduced the migration of damaged DNA in ovarian cancer cells (Fig. 3I J). These data strongly confirmed the role of MRE11:p.K464R mutation in inducing Olaparib resistance in ovarian cancer cells and indicated its potential mechanism to promote tumor cell survival by reducing the accumulation of cellular DNA damage.

### MRE11:p.K464R mutation enhanced the binding ability of MRE11 protein to RAD50/RPS3

**Fig. 4:**
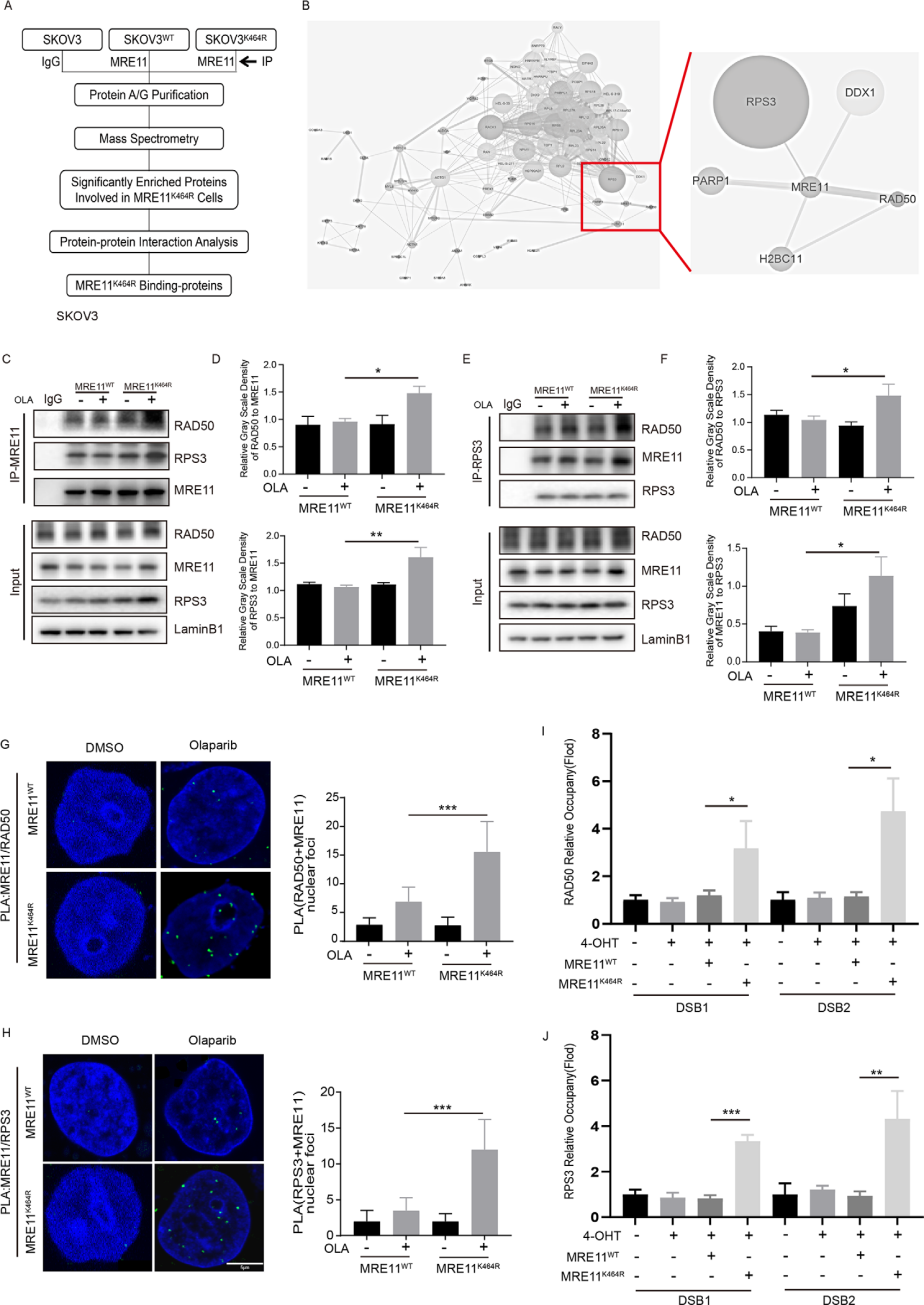
**Interactions of MRE11:p.K464R with RAD50/RPS3**. (**A**) Schematic diagram of IP-MS detection. (**B**) The directly-interacting proteins of MRE11 were screened in the String database according to the IP-MS data. (**C**-**D**) SKOV3- MRE11^WT^/MRE11^K464R^ cells were treated with or without Olaparib for 24 h. The cells were then lysed and immunoprecipitation with MRE11 antibody (**C**) and the relative gray scale density of RAD50(up) and RPS3 (down) to MRE11 are presented in D.(**E**-**F**) SKOV3- MRE11^WT^/MRE11^K464R^ cells were treated with Olaparib or DMSO. The cells were then lysed and immunoprecipitation with RPS3 antibody (**E**) and the relative gray scale density of RAD50 (up) and MRE11 (down) to MRE11 are presented in F. (**G**-**H**) Detection of MRE11-RAD50 (**G**) and MRE11-RPS3(**H**) interaction was carried out by PLA labeling in SKOV3 MRE11^WT^/MRE11^K464R^ cells treated with or without Olaparib for 24 h. Representative images are shown. Scale bars, 5 μm. The scatterplot displays quantification of the PLA signals per nucleus from at least 100 cells from three independent experiments. Data are mean ± SEM. (**I**-**J**) ER-AsiSI Hela cells were transfected with empty vector or MRE11^WT^ or MRE11^K464R^, and then treated with 4-OHT to induce DSBs. RAD50 (**I**) and RPS3 (**J**) accumulation at DNA damage sites generated by AsiSI was detected by ChIP qPCR. Data are presented as mean values ± SEM from three independent experiments. *p < 0.05, **p < 0.01, ***p < 0.001, ****p < 0.0001, ns, not significant, as determined by the unpaired two-tailed Student’s t-test

We further explored the regulatory mechanism underlying the reduction of DNA damage accumulation induced by the MRE11:p.K464R mutation. Starting from the function of MRE11, we identified the highly reliable interacting proteins of MRE11 protein using immunoprecipitation-mass spectrometry (IP-MS) (Fig. 4A) and screened out the proteins that directly bind to MRE11 protein by STRING database [18]. This analysis revealed several proteins, including RAD50, RPS3, PARP1, DDX1, and H2BC11 (Fig. 4B). Furthermore, we clarified the effect of MRE11:p.K464R mutation on enhancing the binding ability of MRE11 to RAD50 and RPS3 through co-immunoprecipitation (Co-IP) assay. We found that when MRE11 was used as the bait protein, the abundance of interacting proteins, including RAD50 and RPS3, was significantly increased in SKOV3 MRE11_K464R mutation group compared with the WT group (Fig. 4C-D). Moreover, increased MRE11 and RAD50 levels were also observed when RPS3 was used as the bait protein for the enrichment of the interacting proteins (Fig. 4E-F). In order to demonstrate the interaction of MRE11:p.K464R and RAD50/RPS3, we conducted proximity ligation assay (PLA) with antibodies targeting MRE11 and RAD50 or RPS3 in SKOV3 MRE11^WT^/ MRE11^K464R^ cells. Compared with MRE11^WT^ cells, PLA signaling significantly increased in MRE11^K464R^ mutation cells after Olaparib treatment (Fig. 4G-H), indicating that the interaction between MRE11 and RAD50/RPS3 was enhanced in MRE11_K464R mutation cells. However, no similar phenotypes were observed for PARP1 and DDX1 (Fig. S3A-B), and the knockdown treatment of two proteins did not significantly affect Olaparib resistance of K464R mutant cells (Fig. S3C). Considering the important roles of RAD50 [28, 29] and RPS3 [30–32] proteins in the process of DNA damage repair, we hypothesize that MRE11:p.K464R mutation may improve the efficiency of DNA damage repair by enhancing the binding of MRE11 protein with RAD50/RPS3 protein.

**Fig. 5:**
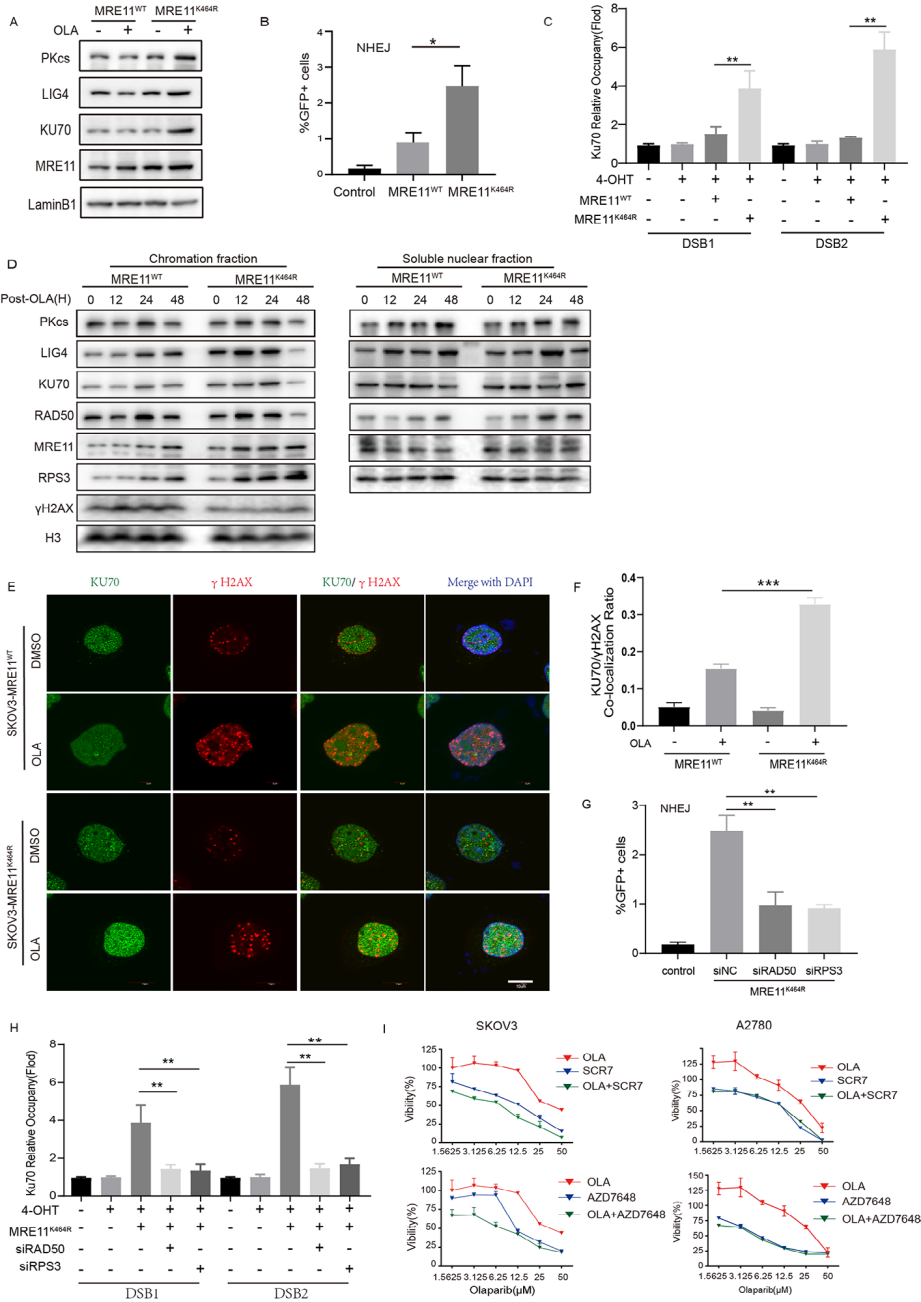
**Effects of MRE11_K464R mutation on NHEJ pathway**. (**A**) Expression of NHEJ key proteins in SKOV3 MRE11^WT^ /MRE11^K464R^ cells after treated with Olaparib for 48 h, detected by Western Blot with indicated antibodies. (**B**) MRE11^WT^ or MRE11^K464R^ virus were infected into EJ5-Hela cells for 24 h. Then, the cells were transfected with an I-SceI expression plasmid for 48 h. NHEJ efficiency were determined by FACS. (**C**) ER-AsiSI Hela cells were infected with MRE11^WT^ or MRE11^K464R^ virus, and then treated with 4-OHT to induce DSBs. Ku70 accumulation at DNA damage sites generated by AsiSI was detected by ChIP qPCR. (**D**) A2780 MRE11^WT^ /MRE11^K464R^ cells after treated with Olaparib for 48 h. The chromatin fractions and the soluble nuclear fractions were analyzed with indicated antibodies by Western Blot. (**E**-**F**) A2780 MRE11^WT^ /MRE11^K464R^ cells were treated with or with Olaparib for 48 h, and co-stained with Ku70 (green) and γH2AX (red) antibodies. E The representative images of immunofluorescence are presented, F quantification of Ku70 and γH2AX co-localization ratio per cell. Each group represents at least 100 cells counted. Scale bar, 10 μm. (**G**) MRE11_K464R with or without siNC/siRAD50/siRPS3 were infected into EJ5-Hela cells for 24 h. Then, the cells were transfected with an I-SceI expression plasmid for 48 h. NHEJ efficiency were determined by FACS. (**H**) MRE11_K464R with or without siNC/siRAD50/siRPS3 were infected into ER-AsiSI Hela cells and then treated with 4-OHT to induce DSBs. Ku70 accumulation at DNA damage sites generated by AsiSI was detected by ChIP qPCR. (**I**) SKOV3 (left) and A2780 (right) MRE11^K464R^ cells were treated for 96 h with indicated doses of Olaparib or SCR7 (up) / AZD7648 (down) alone or combined for 96 h and viability assessed. Data are presented as mean values ± SEM from three independent experiments. *p < 0.05, **p < 0.01, ***p < 0.001, ****p < 0.0001, ns, not significant, as determined by the unpaired two-tailed Student’s t-test

To investigate the response of RAD50/RPS3 to DNA damage and their recruitment to DNA damage sites on chromatin, chromatin fractions of Olaparib treated A2780 MRE11_WT/K464R cells were prepared and analyzed by Western blotting. The results revealed that MRE11, RAD50 and RPS3 accumulated in the chromatin more rapidly after treated with Olaparib in K464R group compared with WT group (Fig. 5D). We also analyzed RAD50 and RPS3 expression in total and nuclear protein after treated with Olaparib and the same effect was observed both in total protein (Supplement Fig. S3E-F) and nuclear (supplement Fig. S3G-H). Notably, the RAD50 protein is mainly expressed in the nucleus [33], and the increased content of RAD50 protein represented the increase of nuclear protein expression. However, the RPS3 protein is widely distributed in cells [34], and the increase of its content of RPS3 may have a potential way to increase the nuclear transport of cytoplasmic RPS3 protein in addition to the increase of nuclear expression [35], and then a greater amount of RPS3 can be recruited to the chromatin more rapidly. Furthermore, to examine whether RAD50/RPS3 is recruited to DNA damage sites, we performed Chromatin immunoprecipitation (ChIP) assays in ER-AsiSI Hela cells. The results showed a significantly higher recruitment of RAD50 and RPS3 to site-specific DNA double-strand break (DSB) sites in the K464R mutant group as compared to the WT group (Fig. 4I-J). And we also used γH2AX as a label for DNA damage sites and RAD50 antibodies to mark the localization of proteins in cells respectively Remarkably, we observed a higher co-localization ratio of RAD50 with γH2AX in the MRE11_K464R group in contrast to the MRE11_WT group (**supplement** Fig. 3I). In summary, MRE11_K464R mutation could recruit more RAD50 and RPS3 to DSB sites, and forming a MRE11-K464R/RAD50/RPS3 complex to accelerate the process of DNA repair, leading to Olaparib resistance.

### Knockdown of RAD50/RPS3 restored the sensitivity of K464R mutant cells to Olaparib

**Fig. 6:**
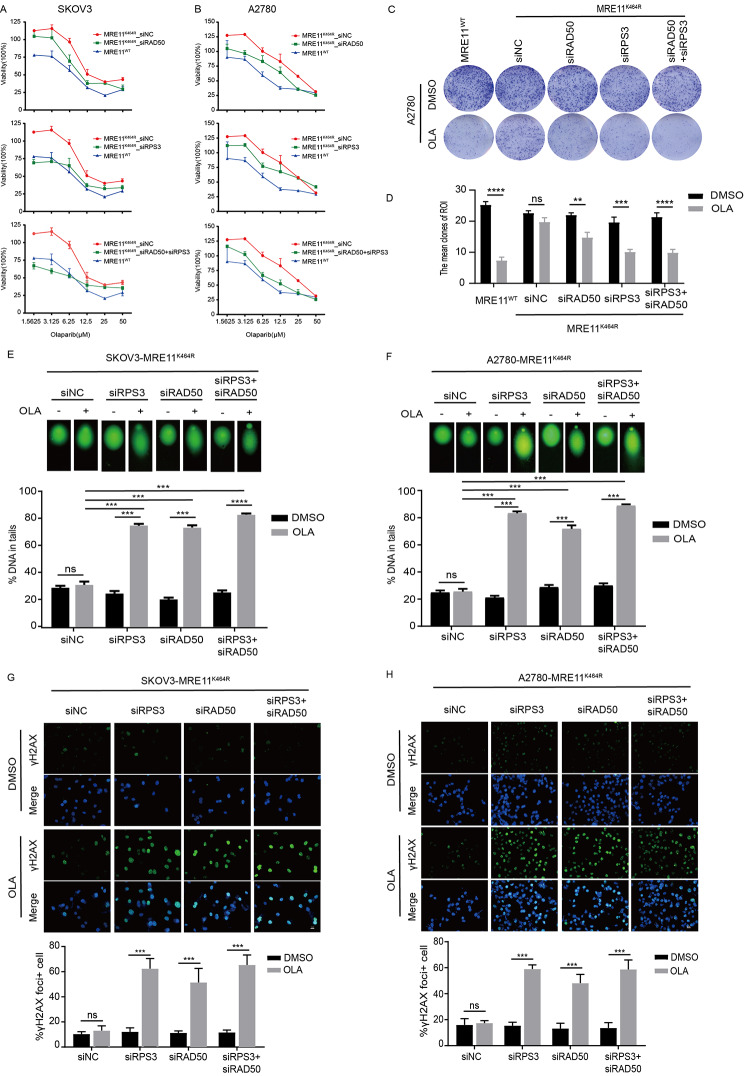
**RPS3/RAD50 knockdown sensitizes K464R mutant cells to Olaparib**. (**A**-**B**) SKOV3 (**A**) and A2780 (**B**) MRE11^K464R^ cells were transfected with siRAD50 or siRPS3 alone or combine for 24 h and then treated for 96 h with indicated doses of Olaparib and viability was measured by CCK8. The expression of scramble siRNA (siNC) was used as control. (**C**-**D**) Cells were transfected with siRAD50 or siRPS3 alone or combine for 24 h and then treated with or without Olaparib for 10 days. The expression of siNC was used as control. Representative pictures of clonogenic assay in A2780 MRE11^K464R^ cells (**C**). The mean clones of ROI are presented (**D**). (**E**-**F**) SKOV3 (**E**) and A2780 (**F**) MRE11^K464R^ cells were transfected with siRAD50 or siRPS3 alone or combine for 24 h and then treated for 48 h and then subjected to Comet analysis. DNA damage is quantified as percent DNA in tails. The expression of siNC was used as control. Each group represents at least 150 cells counted. (**G**-**H**) SKOV3 (**G**) and A2780(**H**) MRE11^K464R^ cells were transfected with siRAD50 or siRPS3 alone or combine for 24 h and then treated with or without Olaparib for 48 h and then stained for γH2AX (green) and DAPI (blue), and γH2AX foci-positive cells were quantified (below). Each group represents at least 150 cells counted. Scale bar, 20 μm. Data are presented as mean values ± SEM from three independent experiments. *p < 0.05, **p < 0.01, ***p < 0.001, ****p < 0.0001, ns, not significant, as determined by the unpaired two-tailed Student’s t-test

The increased RAD50/RPS3 protein in the DNA damage sites on chromatin and their enhanced binding to MRE11 protein mediated MRE11:p.K464R mutation-induced Olaparib resistance. We attempted to explore the possibility of reversing Olaparib resistance in K464R mutant cells by targeting knockdown the RAD50/RPS3 protein. We screened the RNA interference sequences that could effectively reduce the expression of RAD50/RPS3 protein and used them for subsequent drug resistance reversal studies in K464R mutant cells (Fig. S4C). By plotting the resistance curves, we observed the Olaparib resistance induced by MRE11:p.K464R mutation in two mutant cells and the knockdown of RAD50/RPS3 protein partially restored the sensitivity of K464R mutant cells, resulting in a significantly reduced IC50 for Olaparib treatment (Fig. 6A-B). In the colony formation experiment, we also observed that specific knockdown of RAD50/RPS3 protein restored the inhibitory effect of Olaparib on tumor cell proliferation (Fig. 6C-D). Comet assay results also showed that the knockdown of RAD50/RPS3 proteins prolonged the comet tailing, indicating increased DNA damage in the mutant cells (Fig. 6E-F). Moreover, cellular immunofluorescence staining of γH2AX further confirmed that the knockdown of two interacting proteins effectively reversed the Olaparib resistance of the K464R mutant cells, resulting in increased accumulation of intracellular DNA damage (Fig. 6G-H). Interestingly, the combined knockdown of RAD50 and RPS3 did not significantly increase the Olaparib sensitivity of K464R mutant cells compared with RAD50 or RPS3 treatment alone. We speculate that there may be differences in the underlying mechanisms of RAD50 and RPS3 proteins involved in the regulation of MRE11:p.K464R mutation-induced Olaparib resistance. Taken together, the above results clarified the key role of RAD50/RPS3 in the induction of Olaparib resistance by MRE11:p.K464R mutation and revealed the possibility of reversing Olaparib resistance in MRE11:p.K464R mutation carriers by interfering with the expression of the two interacting proteins.

### MRE11:p.K464R mutation enhances NHEJ repair efficiency by restricting RPS3 activity

HR and NHEJ repair are the two main DNA repair pathways [36]. To assess MRE11_K464R increases DNA repair efficiency by HR or NHEJ pathway, we evaluated the expression of relevant proteins after treating with Olaparib in SKOV3 and A2780 MRE11^WT^/ MRE11^K464R^ cells. Interestingly, we observed a significant upregulation of proteins related to the NHEJ pathway (KU70, LIG4 and PKcs) (Fig. 5A; supplement Fig. S4C), while no significant changes were detected in proteins associated with the HR pathway (NBS1, pATM, pATR, pCHEK1 and pCHEK2) (supplement Fig. S4A-B). We also performed NHEJ reporter assays in EJ5-Hela cells, and the results revealed a substantial increase in NHEJ efficiency in the MRE11_K464R mutation cells compared to the WT group (Fig. 5B). The cellular immunofluorescence assay further demonstrated that KU70, LIG4, and PKcs proteins exhibited substantial nuclear aggregation in K464R mutant cells (supplement Fig. S4D-E).

Subsequently, to gain deeper insights into the enhanced NHEJ efficiency in MRE11_K464R mutant cells, we examined the accumulation of NHEJ proteins at chromatin and DSB sites. Chromatin fraction assays revealed that the NHEJ proteins (Ku70, LIG4, and PKcs) can be recruited to chromatin more rapidly in MRE11_K464R mutation group compared with WT group (Fig. 5D). Additionally, the ChIP assays in ER-AsiSI Hela cells also demonstrated a higher accumulation of Ku70 at DSB sites in the MRE11_K464R mutation group (Fig. 5C). Moreover, we used γH2AX to label DNA damage sites and Ku70/LIG4/PKcs antibodies to mark the localization of proteins in A2780 MRE11^WT^/ MRE11^K464R^ cells. The results show that the co-localization ratio of Ku70, LIG4, and PKcs with γH2AX was significantly elevated in the MRE11_K464R group compared to the WT group (Fig. 5E-F; supplement Fig. S5A-B). These findings collectively suggest that Olaparib treatment in K464R mutant cells led to abnormal activation of NHEJ pathway, which mediate the generation of resistance.

To further understand the role of RAD50 and PRS3 in enhancing NHEJ in MRE11_K464R cells, we attempted to explore the possibility of reversing NHEJ pathway in MRE11_K464R mutant cells by targeting knockdown the RAD50/RPS3 protein. First, we tested NHEJ efficiency in EJ5-Hela cells after infected MRE11_K464R with or without RAD50/RPS3-specific siRNA and found that either RPS3 or RAD50 knockdown decreased NHEJ efficiency compared with siNC group (Fig. 5G). The ChIP assays in ER-AsiSI Hela cells showed that the recruitments of KU70 proteins to DNA damage sites was significant decreased after knocking down RPS3 or RAD50 (Fig. 5H). The above results further validate our hypothesis that MRE11-K464R/RAD50/RPS3 may form a more efficient DNA repair complex and accelerate the process of DNA repair by activating NHEJ pathway, leading to Olaparib resistance.

For the abnormal activation of the NHEJ pathway in K464R mutant cells with Olaparib treatment, we evaluated the possibility of reversing Olaparib resistance by intervening in NHEJ pathway. As shown in the results, the application of either DNA-PKcs inhibitor (AZD7648) or DNA Ligase IV inhibitor (SCR7) decreased the IC50 threshold of K464R mutant cells to some extent and increased their sensitivity to Olaparib (Fig. 5I). Unfortunately, the use of the same inhibitors in WT cells did not improve the killing efficacy of Olaparib against ovarian cancer cells, and the IC50 has not been optimized (supplement Fig. S5E-F), but it also further confirmed the leading role of NHEJ repair pathway in Olaparib resistance in K464R mutant cells.

In conclusion, we found and clarified the role of acquired MRE11:p.K464R mutation in inducing Olaparib resistance of patients and further elucidated the underlying mechanism of this mutation mediating abnormal activation of NHEJ repair pathway by enhancing the interaction between MRE11 and RPS3. The results of this study provide a theoretical basis for using the MRE11:p.K464R mutation as an indicator to monitor the resistance in patients with Olaparib maintenance therapy and open up a new idea for exploring the combination therapy of MRE11:p.K464R mutation carriers after Olaparib resistance.

## Discussion

As the core component of MRN complex, MRE11 plays a vital role in the process of DNA double-strand break repair, and the functional variation of MRE11 can significantly influence the efficacy of tumor therapies targeting DNA damage. In previous study, we reported that the acquired high-frequency mutation of MRE11:p.K464R in ovarian cancer patients undergoing Olaparib maintenance therapy, and preliminarily elucidate its correlation with Olaparib resistance [17]. Moreover, we did not find the record of MRE11:p.K464R mutation by searching databases (Cosmic database [37], My Cancer Genome database [38], ICGC database (http://icgc.org), TCGA database (http://www.cancer.gov/ccg/research/genome-sequencing/tcga)), which strongly suggests that acquired MRE11:p.K464R mutation may play a key role in causing Olaparib resistance and may be a specific biomarker for Olaparib and even PARP inhibitor resistance. Therefore, the exploration of the mechanism of resistance caused by MRE11:p.K464R mutation will expand the understanding of PARP inhibitor resistance, and is expected to provide new ideas for the formulation of combination therapy regimens after PARP inhibitor resistance.

In this work, we constructed two human ovarian cancer cell lines containing MRE11:p.K464R mutation based on the clinical facts that acquired MRE11:p.K464R mutation occurred in ovarian cancer patients with Olaparib resistance, and verified the function of this mutation causing Olaparib resistance through different experimental methods. Our findings provide compelling evidence that the MRE11:p.K464R mutation confers increased tolerance to Olaparib and reduces the accumulation of cellular DNA damage in ovarian cancer cells. This is a critical factor in the development of drug resistance in these cells. As we know, the mechanism of PARP inhibitors is to inhibit the PARP enzyme that recognizes and repairs DNA single-strand breaks, resulting in the conversion of SSB to DSB. In cells with defective homologous recombination repair, such as those with BRCA mutations, the accumulation of DSBs caused by PARP inhibitors can induce cell death through synthetic lethality, a promising therapeutic strategy [39]. However, the reduced accumulation of DNA damage caused by MRE11:p.K464R mutation alleviated the stress pressure induced by PARP inhibitors and facilitates the survival of tumor cells, contributing to the development of drug resistance.

Furthermore, we explored the molecular mechanism of MRE11:p.K464R mutation leading to Olaparib resistance. From the perspective of molecular structure, the missense mutation of K464R site is likely to lead to the change of spatial conformation of MRE11, which in turn affects the molecular function of MRE11 protein. Due to MRE11:p.K464R mutation leads to a decrease in DNA damage accumulation, it is strongly suggested that MRE11:p.K464R mutation improves the biological activity of the MRE11 protein and enhances the DNA damage repair. In terms of mechanism of action, the MRE11:p.K464R mutation enhances the binding ability of MRE11 protein to interacting proteins, including RAD50, RPS3, etc., and induces the recruitment of related proteins to DNA damage sites, improving the efficiency of local DNA damage repair. These results are a new exploration of the mechanisms of PARP inhibitor resistance, and also provide a target for the development of related drugs after PARP inhibitor resistance.

The enhanced DNA damage repair observed in the context of MRE11:p.K464R mutation presents a fascinating and intriguing phenomenon. It is generally believed that mutations in genes related to HRR pathway can cause DNA damage repair defects [5], which improve the killing effect of DNA-targeted tumor therapy. The emergence of MRE11:p.K464R mutation leads to the enhancement of DNA damage repair, blocking the accumulation of DNA damage and subsequent tumor cell death, which may lead to the generation of multiple drug resistance of tumor cells, including platinum resistance, PARP inhibitor resistance, antibiotic resistance, etc., suggesting that MRE11:p.K464R mutation may be a common mechanism of resistance to cancer drugs. More interestingly, the enhancement of NHEJ may play a dominant role in MRE11:p.K464R mutation-mediated PARP inhibitor resistance. In previous studies, In previous studies, NHEJ and HRR have been observed to be in a dynamic equilibrium, with repair efficiency being selected during the DNA damage repair process [40–42]. Some studies have shown that the MRN complex promotes DNA end resection primarily through its nuclease activity and guides DNA damage repair to transition towards HRR in the presence of sister chromatin [43–45]. It is worth noting that the MRN complex is also involved in regulating the repair process of NHEJ. The mutations in the MRN dimerization region can impair the efficiency of NHEJ [46], while the deletion of MRN complex subunits also shows a reduction of NHEJ activity [47]. There were also studies prove that Mre11/RAD50 complex (MR) functions as a sensor and coordinator of DSB repair [48], which opens hairpin structures and resect DNA 3’-5’ from the nick. But the internal DNA would need to be bent/melted to fit into the channel, and DNA bending/melting for efficient endonuclease is indeed suggested by biochemical studies [49]. In this study, we found that the MRE11:p.K464R mutation enhanced the binding with RAD50 and RPS3 proteins. It is known that mammalian RPS3 functions as a DNA repair endonuclease [50], subunit with AP lyase activity that can cleave different types of DNA lesions. So, we speculated that the enhanced binding of MRE11 to RAD50 and RPS3 likely contributes to the development of Olaparib resistance by improving NHEJ efficiency.

Considering the decreased activity of HRR pathway and the accompanying abnormal activation of NHEJ, we speculate that the K464R mutant cells reconstructed the dynamic balance of DNA damage repair pathway in response to Olaparib treatment, and selected NHEJ mode to ensure cell survival. And further studies may be needed to elucidate the proportion relationship of repair modes. In addition, as an imprecise repair mechanism, DNA damage repair led by NHEJ may lead to the generation of genomic instability [51]. For example, the participation of X-family DNA polymerase Pol µ can misincorporate dGTP to a certain extent, thereby further activating the downstream reactions of NHEJ and causing genome instability in the repair process of NHEJ [52, 53]. Therefore, this also provides an opportunity for the treatment of ovarian cancer patients with the MRE11:p.K464R mutation. Inevitably, the exploration of intervention methods or combined treatment regimens needs more research data to support them. However, our results strongly suggest that MRE11:p.K464R mutation is an important driving factor of Olaparib resistance, which deserves further exploration of clinical translational potential.

More importantly, the MRE11:p.K464R mutation was rarely reported in previous studies due to the limitations of detection technology. It was only detected in 2 gastric cancer patients’ tissues (the detection rate was 2/66) [54], but there was no in-depth study on this site. Based on the highly specific mutation detection method in the previous study [55], the screened MRE11:p.K464R mutation may bring a breakthrough in the resistance research of PARP inhibitor Olaparib. Admittedly, we need to acknowledge that there may be differences in the resistance mechanisms among PARP inhibitors, and the MRE11:p.K464R mutation may not be the only driver of resistance. However, the discovery of MRE11:p.K464R mutation provides a feasible reference index for resistance monitoring of Olaparib, and it is expected to extend to other PARP inhibitors, or to expand the combination therapies.

In conclusion, our study elucidates the function and underlying mechanism of MRE11:p.K464R mutation leading to Olaparib resistance, and provides a potential target for the formulation of combination therapy regimens after drug resistance. More importantly, our study provides a theoretical basis for MRE11:p.K464R mutation as a specific indicator for resistance monitoring of Olaparib, which is expected to optimize the application performance of PARP inhibitors and achieve more accurate treatment and prognosis for ovarian cancer.

### Electronic supplementary material

Below is the link to the electronic supplementary material.

Supplementary Material 1

Supplementary Material 2

Supplementary Material 3

Supplementary Material 4

Supplementary Material 5

Supplementary Material 6

Supplementary Material 7

Supplementary Material 8

Supplementary Material 9

Supplementary Material 10

## Data Availability

Not applicable.
