# Supplementary figures and images for: MRE11:p.K464R mutation mediates olaparib resistance by enhancing DNA damage repair in HGSOC
Source: Cell Biosci. 2023 Sep 27;13:178. doi: 10.1186/s13578-023-01117-0 (PMC10537967; doi:10.1186/s13578-023-01117-0)

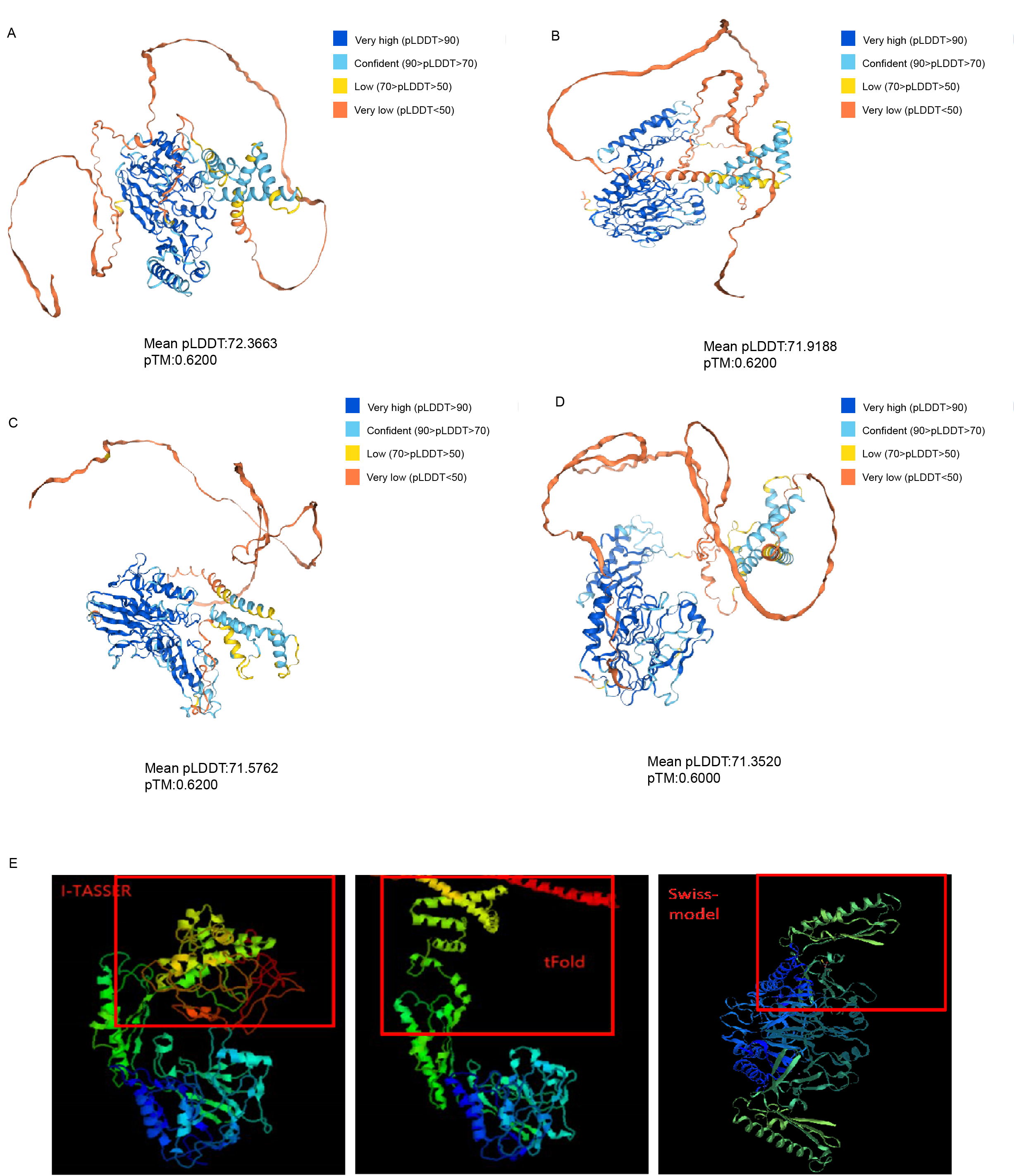

Supplement: Supplementary file 1 — Supplementary Material 1 [file 13578_2023_1117_MOESM1_ESM.png]

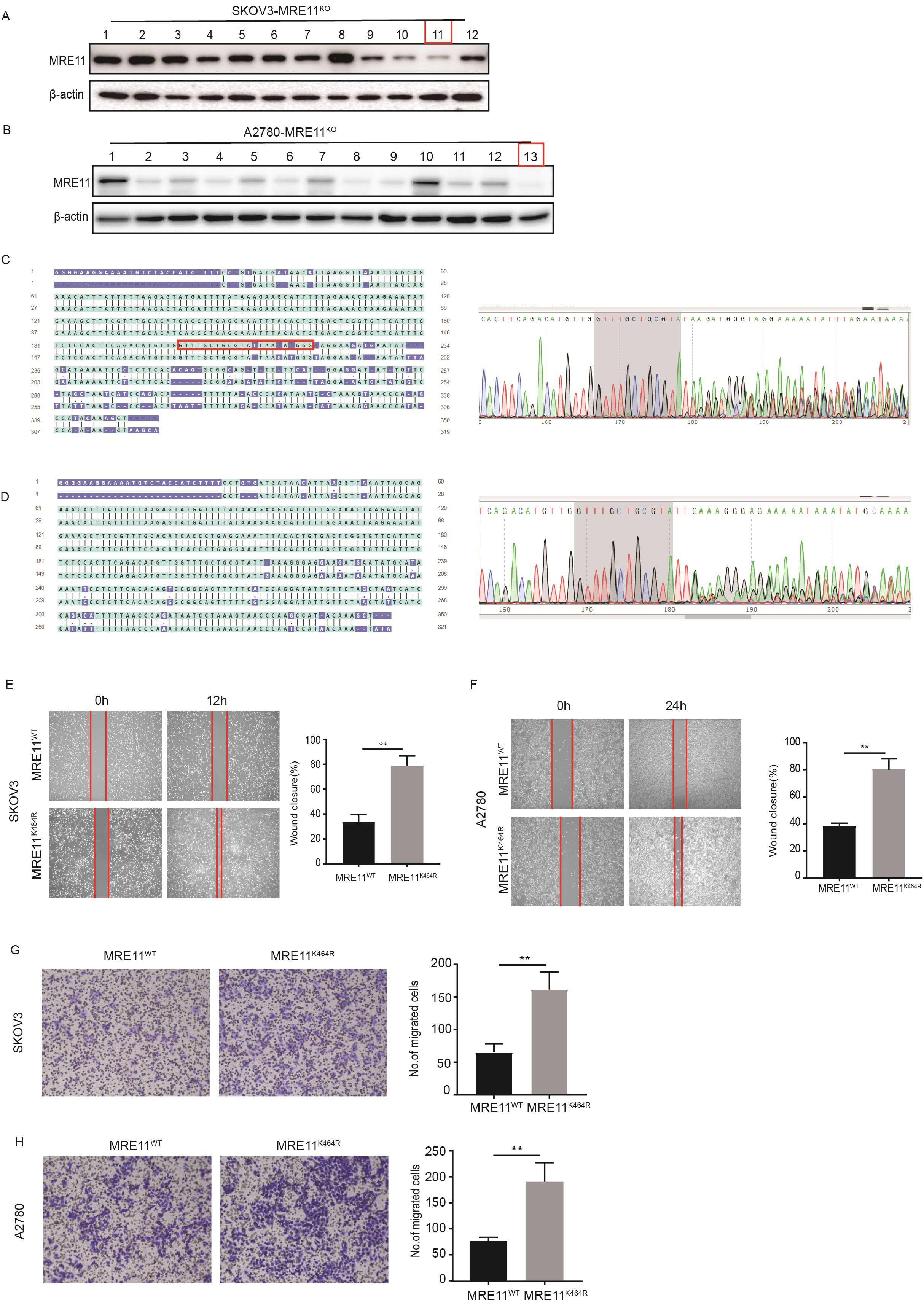

Supplement: Supplementary file 2 — Supplementary Material 2 [file 13578_2023_1117_MOESM2_ESM.png]

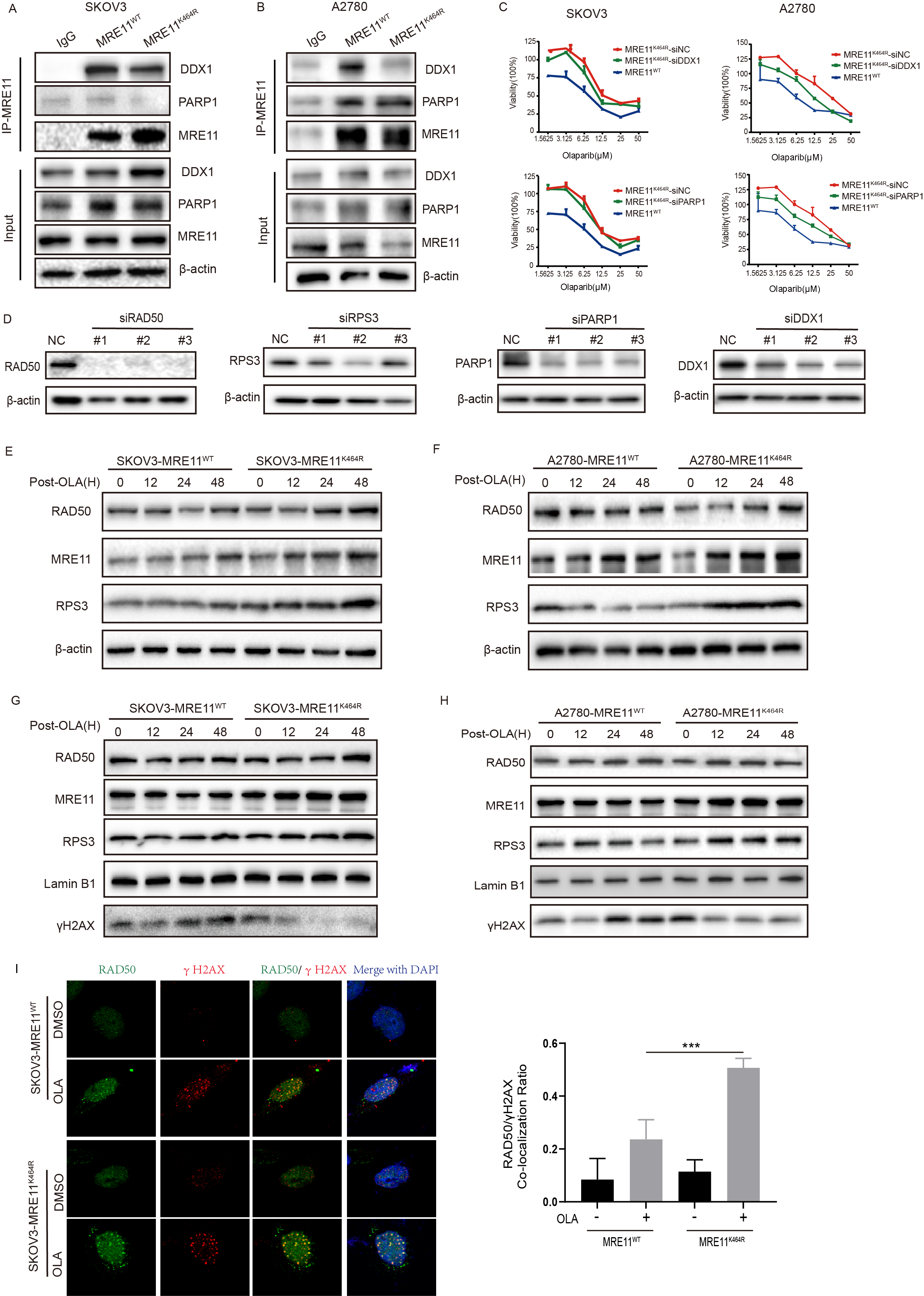

Supplement: Supplementary file 3 — Supplementary Material 3 [file 13578_2023_1117_MOESM3_ESM.png]

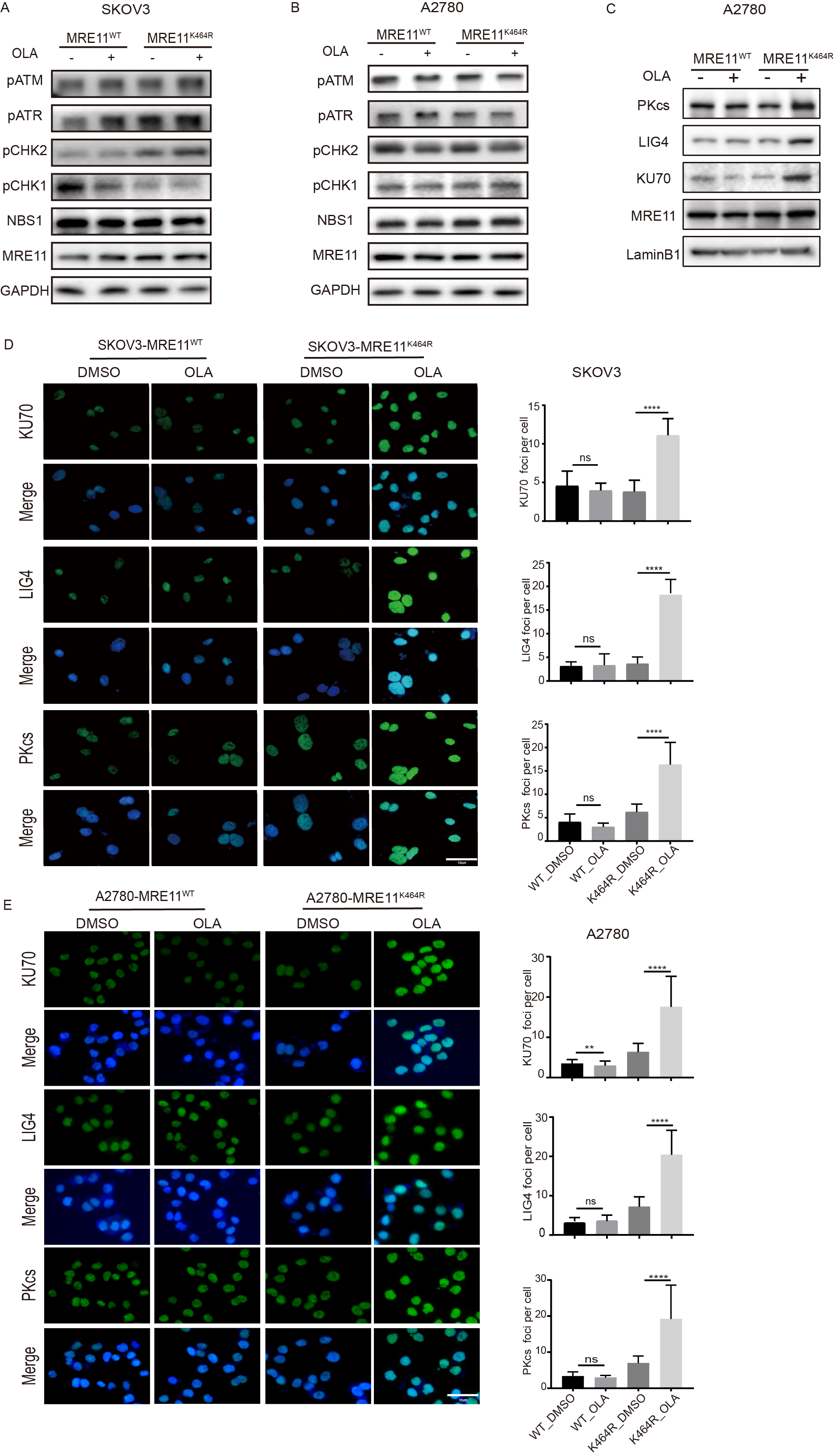

Supplement: Supplementary file 4 — Supplementary Material 4 [file 13578_2023_1117_MOESM4_ESM.png]

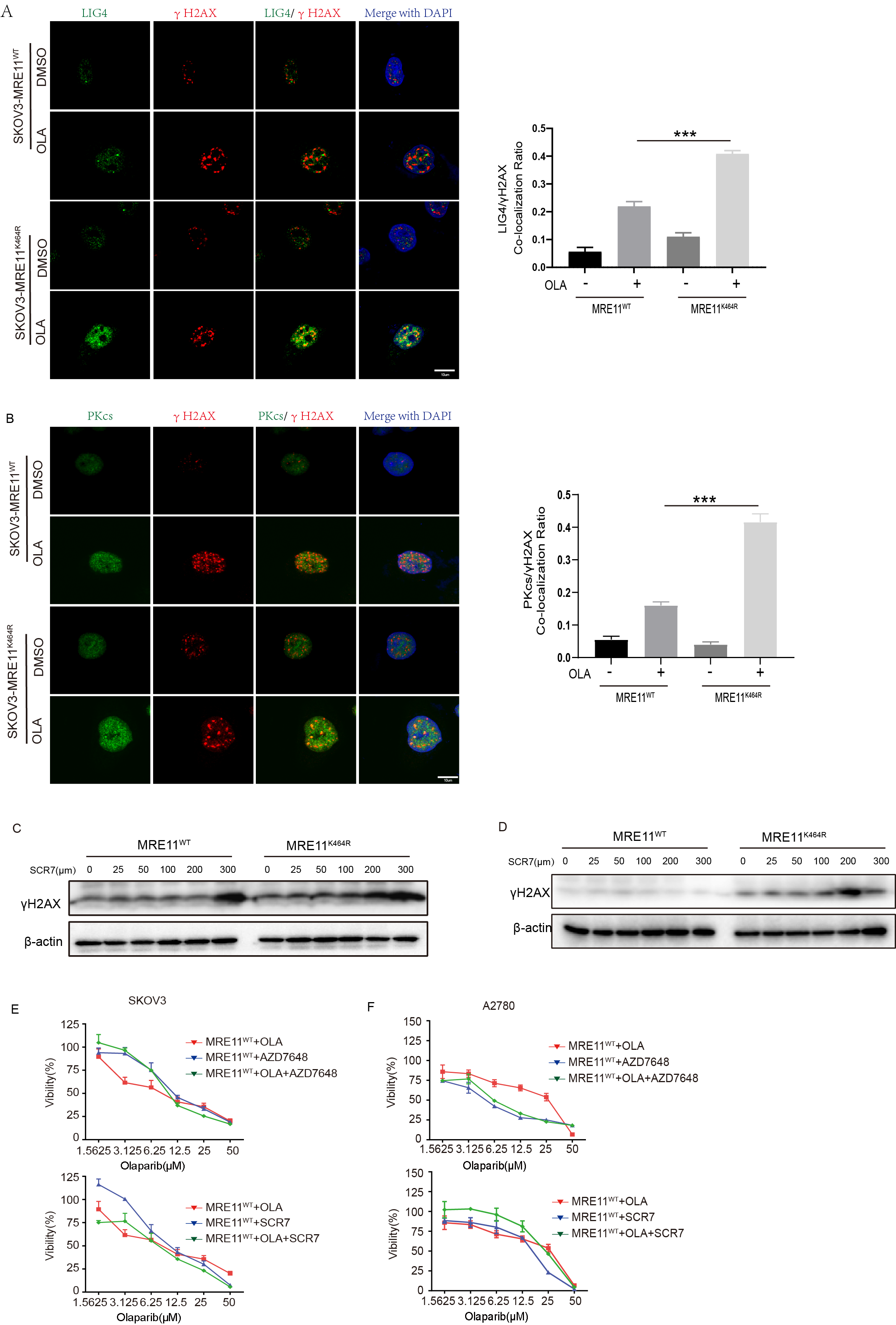

Supplement: Supplementary file 5 — Supplementary Material 5 [file 13578_2023_1117_MOESM5_ESM.png]
