# Supplementary material for: MRE11:p.K464R mutation mediates olaparib resistance by enhancing DNA damage repair in HGSOC
Source: Cell Biosci. 2023 Sep 27;13:178. doi: 10.1186/s13578-023-01117-0 (PMC10537967; doi:10.1186/s13578-023-01117-0)
Supplement: Supplementary file 9 — Supplementary Material 9 [file 13578_2023_1117_MOESM9_ESM.doc]

**Supplemental Information**

**Fig_S1 Predicted 3D structure of K464R mutation** (A-D) The predicted 3D structure based on the Rank 2-5 model confidence (pLDDT). (E) The prediction of the three-dimensional structure of MRE11:p.K464R protein using I-TASSER, tFold and Swiss-model, respectively.

**Fig_S2 Effects of K464R mutation on migration and invasion function of ovarian cancer cells.** (A-B) Western blot assay showing gene silencing by CRISPR-Cas9 technology mediated MRE11-knockout (MRE11^KO^) in SKOV3 (A) and A2780 cells (B). SKOV3-MRE11^KO^ clone@11 and A2780-MRE11^KO^ clone@13 was selected for further study. (C-D) The DNA sequence of the knockout region by sanger sequencing of SKOV3-MRE11^KO^ clone@11 (C) and A2780-MRE11^KO^ clone@13(D), respectively. The position of sgRNA was highlight by red frame line. (E-F) Representative images of wound-healing assay of SKOV3 (E) and A2780 (F) MRE11^WT^/MRE11^K464R^ cells. Wound healing rates are displayed on the lower. (G-H) Representative images of transwell assay of SKOV3 (E) and A2780 (F) MRE11^WT^/MRE11^K464R^ cells are on the left. Data are presented as mean values ± SEM from three independent experiments. *p < 0.05, **p < 0.01, as determined by the unpaired two-tailed Student’s t-test.

**Fig_S3 Interactions of MRE11:p.K464R with DDX1/PARP1.** (A-B) MRE11 and DDX1/PARP1 interaction was not significantly increased in SKOV3 (A) and A2780-MRE11^K464R^ (B) cells compared with the WT group by immunoprecipitation with MRE11 antibodies. (C) SKOV3 (left) and A2780 (right) MRE11^K464R^ cells were transfected with siNC or siDDX1 (up) / siPARP1 (down) for 24 h and then treated for 96 h with indicated doses of Olaparib and viability assessed by CCK8. (D) Western blot analysis showing gene silencing by three siRNAs against RAD50/RPS3/PARP1/DDX1 for 48 h in SKOV3. The expression of scramble siRNA (siNC) was used as control, and siRAD50#1, siRPS3#2, siPARP1#2 and siDDX1#3 was used for further study. (E-H) SKOV3 and A2780 MRE11^WT^/MRE11^K464R^ cells were treated with Olaparib and then lysed immediately (0 h) or treated for the indicated time and total protein (E-F) and nucleoprotein (G-H) were analyzed by Western blot with the indicated antibodies. (I) SKOV3 MRE11^WT^/ MRE11^K464R^ cells were treated with or with Olaparib for 48 h, and co-stained with RAD50 (green) and γH2AX (red) antibodies. The representative images of immunofluorescence are presented at right, and quantification of RAD50 and γH2AX co-localization ratio per cell are presented at left. Each group represents at least 100 cells counted. Scale bar, 10μm. Error bars represent the SEM of the mean (n = 3). ****p < 0.0001, ns, not significant, as determined by the unpaired two-tailed Student’s t-test.

**Fig_S4 Effects of MRE11_K464R mutation on expression of HRR and NHEJ pathway proteins.** (A-B) SKOV3 (A) and A2780 (B) MRE11^WT^/MRE11^K464R^ cells were treated with or without Olaparib and harvested after 48 h cultivation. The level of key proteins in the HRR pathways was determined by Western Blot with indicated antibodies. (C) Expression of NHEJ key proteins in A2780 MRE11^WT^ /MRE11^K464R^ cells after treated with Olaparib for 48 h, detected by Western Blot with indicated antibodies. (D-E) SKOV3 and A2780 MRE11^WT^/MRE11^K464R^ cells were treated with or without Olaparib for 48 h, and stained with Ku70, LIG4 or PKcs antibody. Representative images of immunofluorescence in SKOV3 (D) and A2780 (E) MRE11^WT^/MRE11^K464R^ cells are in left and quantification of Ku70, LIG4 or PKcs foci per cell in right. Each group represents at least 100 cells counted. Scale bar, 10μm. Error bars represent the SEM of the mean (n = 3). ****p < 0.0001, ns, not significant, as determined by the unpaired two-tailed Student’s t-test.

**Fig_S5 MRE11_K464R mutation recruits NHEJ pathway proteins to DSB sites. (**A) A2780 MRE11_WT/K464R cells were treated with or with Olaparib for 48 h, and co-stained with LIG4 (green) (A) or PKcs (green) (B) and γH2AX (red) antibodies. The representative images of immunofluorescence are presented at left, and quantification of LIG4 or PKcs and γH2AX co-localization ratio per cell are presented at right. Each group represents at least 100 cells counted. Scale bar, 10 μm. (C) SKOV3 (D) and A2780 (E) MRE11^WT^/MRE11^K464R^ cells were treated with different concentration SCR7 and then lysed after treated with 48 h and proteins were analyzed by Western blot with the indicated antibodies. (E) SKOV3 (left) and A2780 (right) MRE11^WT^ cells were treated for 96 h with indicated doses of Olaparib or AZD7648 (up) / SCR7 (down) alone or combined for 96 h and viability assessed. Data are presented as mean values ± SEM from three independent experiments. *p < 0.05, **p < 0.01, ***p < 0.001, ****p < 0.0001, ns, not significant, as determined by the unpaired two-tailed Student’s t-test.
