# Supplementary material for: MRE11:p.K464R mutation mediates olaparib resistance by enhancing DNA damage repair in HGSOC
Source: Cell Biosci. 2023 Sep 27;13:178. doi: 10.1186/s13578-023-01117-0 (PMC10537967; doi:10.1186/s13578-023-01117-0)
Supplement: Supplementary file 10 — Supplementary Material 10 [file 13578_2023_1117_MOESM10_ESM.docx]

**Table_S1 Sequences of siRNA used in this study**

|  | sense（5'-3'） | antisense（5'-3'） |
| --- | --- | --- |
| Human-siRAD50#1 | GUGAUUUAGACAGGCUUAAdTdT | UUAAGCCUGUCUAAAUCACdTdT |
| Human-siRPS3#2 | GCAGAGUCUCUGCGUUACAdTdT | UGUAACGCAGAGACUCUGCdTdT |
| Human-siDDX1#2 | GGGCAAUCAAGGAACAUAAdTdT | UUAUGUUCCUUGAUUGCCCdTdT |
| Human-siPARP1#3 | CCAAAGGAAUUCCGAGAAAdTdT | UUUCUCGGAAUUCCUUUGGdTdT |
| DSB1 | GATTGGCTATGGGTGTGGAC | CATCCTTGCAAACCAGTCCT |
| DSB2 | TTCCTGCAGCCTCATTTTCT | TGATGATGCCTTTTCCCTTC |
